# A report on parent involvement in planning a randomised controlled trial in neonatology and lactation – insights for current and future research

**DOI:** 10.1186/s13006-022-00509-1

**Published:** 2022-09-14

**Authors:** Ilana Levene, Fiona Alderdice, Beth McCleverty, Frances O’Brien, Mary Fewtrell, Maria A. Quigley

**Affiliations:** 1 National Perinatal Epidemiology Unit, Nuffield Department of Population Health, University of Oxford, Oxford, UK; 2 Bliss, Maya House, 134-138 Borough High Street, London, UK; 3 Newborn Care, John Radcliffe Hospital, Oxford University Hospitals NHS Trust & Faculty of Clinical Medicine, University of Oxford, Oxford, UK; 4 UCL Great Ormond Street Institute of Child Health, London, UK

**Keywords:** Public engagement, Breastfeeding, Prematurity, Human milk

## Abstract

**Background:**

Patient and Public Involvement (PPI) is a rich and valuable part of the process of planning, designing, carrying out and disseminating research. It is important to communicate PPI findings in detail so that the contributions of those involved are fully utilised and disseminated. The extended and iterative PPI process used within a neonatal randomised controlled trial related to the expression of breastmilk after very preterm birth is reported here.

**Methods:**

Seven iterative stages of PPI were used. Stage 1 was informal PPI using historical interaction with parents and publicly available resources. Stage 2 was an online questionnaire open to parents of premature babies and advertised via a charity collaborator. Stage 3 was partnership with a charity collaborator. Stage 4 was a set of online panels focusing on study design and documents. Stage 5 was an interactive exercise to modify the trial intervention. Stage 6 is the presence of PPI contributors on the trial steering committee. Stage 7 is a dissemination panel. At each stage attention was paid to the diversity of participants involved, with strategies to increase the involvement of parents from under-reached groups.

**Results:**

Six hundred and seventy-five participants responded at Stage 2, six parents were involved at Stage 4 and 12 parents at Stage 5. PPI contributed to the choice of study question, outcomes and produced a set of questions for future research. PPI impacted on the study design, with specific emphasis on reducing participant distress related to lactation, and reducing the burden of being involved in research at a time of significant stress.

**Conclusions:**

PPI had a far-reaching influence on this neonatal randomised controlled trial during the planning and design phase, which reinforces the importance of PPI at the earliest stages of the research cycle. The online questionnaire format elicited an unexpectedly deep and broad pool of transferable insights, which will have an impact on future research focus and design in the area of lactation and prematurity. Approaches to increasing PPI involvement from under-reached populations are important and can be successful despite resource constraints.

**Supplementary Information:**

The online version contains supplementary material available at 10.1186/s13006-022-00509-1.

## Background

### What is PPI?

Patient and Public Involvement (PPI) is the process of involving those who benefit from research in planning, performing and disseminating research. In a neonatal context, the term patient covers the parents and family members of a newborn as well as older children or adults who experienced neonatal conditions [1]. PPI at every stage of research is becoming more established [2, 3], but brings with it a complex power dynamic and a need for specific resource allocation at any early stage, often before funding applications are written [4]. Early stage researchers, particularly students, may therefore encounter challenges to integrating effective PPI into research plans [5].

Positive impacts of PPI include increasing trial recruitment or retention [2], refining the research question to ensure it has maximum utility [6], correcting bias and incorrect assumptions [7], and pre-emptively identifying potential problems [8]. In addition, PPI can be viewed through a rights-based lens as a social good [6] and through a political lens as necessary for the accountability and legitimacy [9] of science. It is important to pay attention to potential discrimination in whose voices are heard, which will involve particular emphasis on involving marginalised groups [6, 10, 11].

### Trial context

This paper reports the PPI process associated with designing a neonatal randomised controlled trial (RCT) in the field of breastmilk feeding after a very preterm birth (less than 32 weeks of completed gestation). Complications arising from preterm birth are the leading cause of neonatal death globally [12]. Babies born very preterm have higher mortality rates through infancy and beyond [13], as well as increased rates of disability [14]. Increasing the amount of mother’s own milk (MOM) given to very preterm babies is an important intervention to lessen morbidity and mortality, particularly in relation to necrotising enterocolitis, a serious gut condition [15]. However very preterm babies cannot directly breastfeed due to their immaturity, so mothers must establish and then maintain their breastmilk supply by mechanical expression, which is challenging and anxiety provoking [16, 17]. Mothers of very preterm babies have a high risk of low milk supply [18–22]. The RCT will test a relaxation intervention for mothers and assess its impact on breastmilk feeding and mental health outcomes. The term breastmilk feeding refers to provision of breastmilk to the infant by any route (for example gastric tube, cup, bottle or direct breastfeeding).

### Reporting PPI

When significant resources have been devoted to PPI and new insights gained, it is important that the PPI process is reported in detail [23] so that the contributions of those involved are fully utilised and disseminated to other researchers, avoiding ‘invisible’ or ‘black box’ PPI that wastes the time and efforts of PPI collaborators [24]. PPI reports also stimulate researchers to challenge their own practice, which can be tokenistic [4, 25], and can share lessons learned, which are common and transferable [4]. PPI reports should cover short term outcomes (what was said and learnt), medium term outcomes (changes resulting from this learning) and long term impacts, for example on the future research agenda, as these can all be used by the wider research community [7]. Such reports are becoming more frequent [23, 26, 27].

This report aims to examine the short, medium and long-term impacts of an extended PPI process in the design of a neonatal RCT, providing useful insights for the design and structure of future research.

### Overview of PPI stages

**Fig. 1:**
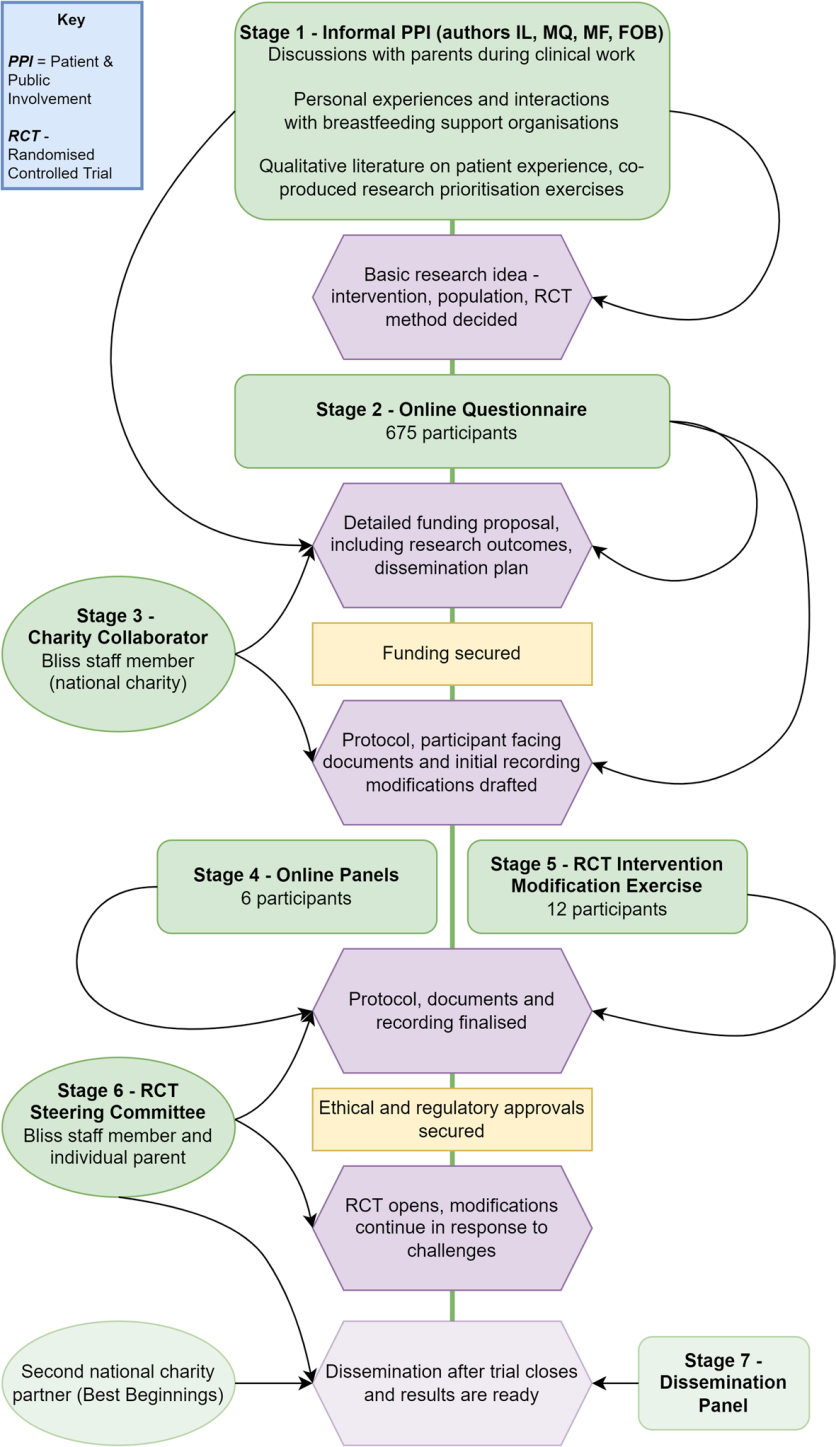
Interaction of PPI stages with the research lifecycle, including future plans

There were five major PPI stages at the planning and design stage of the RCT (stage one to five), one stage supports the ongoing trial management (stage six) and one further stage is planned for dissemination (stage seven). These stages are described in Table 1 and their relationship with the trial lifecycle is shown in Fig. 1. This paper will focus on stages one to five as these are the stages that are often missing from PPI implementation [4] (See Table 1).

**Table 1:** Description of PPI stages complete, ongoing and planned

Stage	Description	Demographic characteristics
1. Informal PPI (complete)	Informal discussions with families over ten years of neonatal practice; personal experiences with expressing breastmilk and breastfeeding support communities; published qualitative literature and partnership prioritisation exercises	Varied family characteristics. Four of the authors involved at this stage had experiences related to expressing milk.
2. Online Questionnaire (complete)	Online questionnaire with free text questions and prioritisation exercises. Advertised via Bliss (national charity for sick and preterm infants)	675 contributors. 71% experienced birth before 32 weeks of pregnancy. 95% white ethnicity
3. Charity Collaborator (complete)	Bliss staff members involved in creation of funding application and RCT protocol	Two staff members
4. Online Panels (complete)	Two evening online panels. Contributors sent documents to review in advance. Discussion of trial design and documents encouraged. Role description provided in advance and payment offered as per INVOLVE guidance [27]	Six contributors. Two from Black and other minority ethnic groups. One was under 25 years old
5. RCT Intervention Modification Exercise (complete)	Contributors sent two draft intervention recordings. Focused questionnaires with feedback and recommendations. Role description provided in advance and payment offered as per INVOLVE guidance	12 contributors. Four from Black and other minority ethnic groups. Two contributors with English as a second language. One was under 25 years old
6. RCT Steering Committee (ongoing)	PPI contributors on the Trial Steering Committee attend meetings and are consulted when difficulties occur. Role description provided in advance and payment offered as per INVOLVE guidance.	One parent and one Bliss staff member
7. Dissemination Panel (planned)	A PPI dissemination panel will be formed to discuss strategy and create resources. Best Beginnings (a national charity) will co-create printed resources for neonatal units	Study participants who consented for further contact will be invited

Figure 1 shows that early PPI can influence the entire research cycle, and that later PPI stages can deepen and refine elements that have been raised in earlier, broader stages. It also illustrates the practical problem that early PPI occurs before trial funding is obtained, and must therefore be performed with minimal resource or using alternative funding strategies [4] (See Fig. 1].

## Integrated methods and results: structure and influences of PPI stages 1 to 5

### Stage 1 – informal PPI

#### Structure

The primary researcher (IL) is a neonatal clinician who has listened to parents of preterm babies during clinical work over ten years of practice, with a particular interest in lactation. IL also has personal experience of expressing breastmilk for prolonged periods and extensive involvement in breastfeeding support organisations and peer support networks as a service user and volunteer.

Qualitative literature and published PPI were used as another way to hear the voice of parents of very preterm infants [16, 17, 26, 28–32] and co-produced research prioritisation exercises [33] were consulted. All of these factors inform an understanding of parental experiences, priorities and values that can be used as a springboard for formal PPI planning.

#### Influence

This informal PPI foundation influenced decisions about the intervention area (relaxation recording), the population (mothers of very preterm babies), the method (RCT) and the broad outcome set (relating to expressed milk volume and breastfeeding experiences).

### Stage 2 – online questionnaire

#### Structure

An online questionnaire was advertised through social media channels of Bliss, a large charity supporting families with sick and preterm babies in the United Kingdom. Bliss provides parent and professional facing resources, individual support, national advocacy and supports research. The questionnaire was designed to elicit opinions from parents of preterm infants with experience of lactation on research priorities related to lactation, what outcomes are most important, potential practical and ethical difficulties of a trial and where parents obtain lactation information. All contributors were sent an executive summary of the findings of the questionnaire as feedback. No specific target number of respondents had been pre-specified however the questionnaire was closed after 1 week due to the overwhelming response.

Six hundred and seventy-five people responded to the questionnaire (respondents were not asked their gender but all had experience of lactation). Forty-seven percent had an infant born between 28 and 32 weeks of gestation (“very preterm”). A quarter (24%) had an infant born at less than 28 weeks’ gestation (“extremely preterm”). Half (51%) had provided breastmilk to their infant for 6 months or less. A quarter (23%) had provided breastmilk for more than 1 year. 95% of respondents reported white ethnicity. The sample included 30 mothers from an ethnic minority background and 52 who were under 25 years old at the time of the preterm birth.

#### Influence – choice of outcomes

The first key influence on the research design was in the choice of outcomes. When asked what questions they had about how to optimally express for and breastfeed a preterm baby, the most common question was how to express more milk and one in five respondents raised this in their free text response (21%). When asked about lactation problems, 27% reported that they had low milk supply and/or could not express as much milk as they wanted. This focus on milk volumes was seen in all groups, including younger mothers and those from ethnic minorities.

Respondents were asked to rank seven pre-defined research outcomes identified through the Stage 1 PPI process. Three outcomes were dominant; being able to express more milk (top priority for 32%), being able to breastfeed for longer (top priority for 25%) and feeling that milk supply was more secure (top priority for 19%).

These results gave a strong direction that the primary outcome of the study should be expressed milk yield, and that duration of breastmilk feeding was an important secondary outcome.

Although milk yield was dominant when asked about problems experienced and questions they would like answered, it was not a dominant response when asked what success or improvement meant in the area of lactation, there was very little consensus on this issue and no single type of response was given by more than 10% of respondents. Table 2 shows the diversity of responses (See Table 2).

**Table 2:** Examples of the diverse range of answers to questions about what ‘success’ means to parents in relation to breastmilk feeding in prematurity

Answers relating to the nature of the breastmilk feeding experience	Answers related to the duration of breastmilk feeding
Being able to express more milk	For longer than actually experienced
Minimising the effect of expressing on time spent with the baby	Until complementary feeds (“solids”) were introduced
Providing exclusive maternal milk	Until discharge from hospital
Any direct breastfeeding (getting the baby to latch to the breast at all)	Until the baby was perceived as less vulnerable to infection (for example after winter or when the baby had reached a particular weight)
Exclusive direct breastfeeding	Six months from birth
The mother being more happy or relaxed in relation to lactation	A year from birth
Feeling well supported	Until the baby was a toddler
Improving the baby’s weight gain	Until the baby or child decided they wanted to stop breastfeeding
Feeling listened to and trusted by staff	

The stress experienced in relation to lactation was evident from the language used by respondents. Frequently used descriptive words were “struggle” (used 87 times), “problem” (81 times), “difficult” (72 times), “stress” (60 times), “hard” (50 times) and “pressure” (24 times). Descriptions of stress and impact on mental health often revolved around milk volumes and milk supply.

***“Constantly anxious about expressing enough milk”.***

***“[pumping] added to my post-natal trauma and depression. I will never forget how much of a failure***. ***.. I felt.”***

***“I was heartbroken that I couldn’t do the one thing I thought I should have been able to do!”***

This wide variety of issues raised, including mental health, expressing efficiency and duration of breastfeeding, informed the choice of secondary outcomes. As anxiety was the overwhelming emotion expressed in relation to expressing milk it was a clear choice for psychometric assessment. It was clearly important to many families to breastmilk feed beyond discharge, whereas discharge is often used as a surrogate marker of breastmilk feeding success due to the relative ease of measurement. However, there was no agreement on a single definition of successful breastfeeding for preterm babies; decision making around duration encompassed not only limitations experienced (such as milk supply or growth pattern), but also parental perceptions of the baby’s medical status and fragility, previous plans and the conflicts experienced between lactation plans and mental health. Therefore, the research design incorporated established public health goals, any breastmilk and exclusive breastmilk, at time points that were logistically or physiologically relevant. The first timepoint chosen was 36 weeks’ post-menstrual age (4 weeks before the estimated date of delivery), as a standardised timepoint commonly used in neonatal research. The second timepoint chosen was 4 months’ corrected age (4 months after the estimated date of delivery), as a time point representing the likely maximum milk supply required and an age where complementary feeds are indicated in almost all cases for very preterm babies [34]. It was also decided to ask participants for their breastmilk feeding goals at baseline to inform subgroup analysis.

#### Influence - addressing anxiety over milk yield in study design

A third of respondents (29%) reported at least one concern about the idea of a research study looking at expressing and breastfeeding in preterm babies. The predominant concern was that many mothers have feelings of guilt, judgement, inadequacy and anxiety related to lactation and that being in a study where volumes of expressed milk are monitored and questions asked about breastfeeding and mental health outcomes could exacerbate this, increasing a feeling of pressure or trauma.

Respondents made suggestions to mitigate these risks, which included:

- Communicate sensitively, respectfully and tactfully
- Ensure that families do not feel pressured over breastmilk outcomes but give gentle encouragement, reassurance, support and empowerment
- Be explicit that many mothers have difficulty expressing and breastfeeding and this is ‘ok’
- Minimise face to face questions as this could impose more pressure and guilt than filling in details electronically
- Ensure mothers feel heard

These responses were used to draft the participant facing documents, using appropriate language to reduce any feeling of pressure and ensure that participants were aware that some people can find it hard to express milk. They also supported the decision to predominantly use a more impersonal electronic interface rather than face-to-face data collection.

#### Influence – minimising burden for participants

Some respondents reported a concern that the research study would be an extra burden to participants, taking time away from their baby and intruding into their lives at a stressful time. They recommended making the process as easy as possible to minimise this burden. Expressing milk was clearly already a considerable burden that was in direct competition with maternal needs, including factors that might be protective of their mental health such as sleep and time with family, so adding an extra component of yield measurement was a concern.

***“. .. often a choice between sleeping and expressing which leads to horrendous guilt when choosing the former and risking health to prioritise the latter”.***

***“Expressing was just difficult due to the time spent between hospital/home/school run/eating, sleeping and showering!”***

In response to these concerns, milk yield monitoring was minimised to three or four time points (depending on gestation at birth), rather than a daily log as used in some studies. Study design was built around electronic data entry, with participants receiving a personalised website link to enter data, in an attempt to make the process as quick and easy as possible. Text message and email reminders were automated for additional efficiency.

A tension exists between the desire for high accuracy assessment of milk yield, using weight, and the burden on participants, where reading the volume of milk from the container would impose less burden than using a weighing scale. A decision was made to prioritise the use of weights for accuracy while also minimising the number of timepoints that participants are asked to do this. However, it remained clear through the trial that weighing milk was difficult for some participants and for future trials, volume reporting may be preferable where medical grade containers with appropriate volume markings are available. It may also be preferable for research staff to measure milk yield rather than participants, although this could interfere with milk provision for the baby unless trained staff are available at all times.

An area of tension between scientific priorities and the burden on parents at a time of heightened stress was the timing of recruitment. PPI contributors raised concerns that recruiting in the first days after preterm birth would cause distress. However, there are strong physiological reasons to suspect that interventions to improve milk supply need to occur as early as possible after birth [35–37]. Many neonatal trials recruit participants in the first days after birth [38] and some in the first hours after birth where this is required due to a need for urgent action [39]. A decision was made to allow recruitment up to day four after birth to balance these factors.

#### Influence – additional data analysis

Contributors had many questions about milk expression, summarised in Table 3 in order of frequency raised. More detail is available in supplementary Table 1 (see Additional file 1). These questions influenced the choice of outcomes for a planned secondary analysis of the data gathered in the RCT, and is also a source of influence for planning future research (See Table 3).

**Table 3:** Questions for future research on breastmilk expression, from PPI contributors, in order of frequency mentioned

How to increase or maintain milk yield (*n* = 87; including food, drink, medication, herbal remedies (*n* = 17), methods focusing on connection to the baby (*n* = 8) and mechanical methods (*n* = 23))	
How frequently to express (*n* = 50)	
How long to express for at each session/optimal pattern of expressing (*n* = 30)	
How best to relax/feel comfortable or happy while expressing milk (*n* = 25)	
How much milk should be targeted/expected (*n* = 23)	
How best to store breastmilk (*n* = 11)	
The impact of delayed first expression/best timing (*n* = 10)	
How to minimise and manage over-supply (*n* = 9)	
Personalised likelihood of being able to express good volumes of milk (*n* = 9)	
The need for and impact of sterilisation processes for pump equipment (*n* = 7)	
How to reduce the occurrence of blocked milk ducts and mastitis (*n* = 4)	
Personalised expectations for the time for milk to ‘come in’ (lactogenesis II) (*n* = 4)	
The impact of antenatal expressing (*n* = 3)	

#### Influence - dissemination plans

**Fig. 2:**
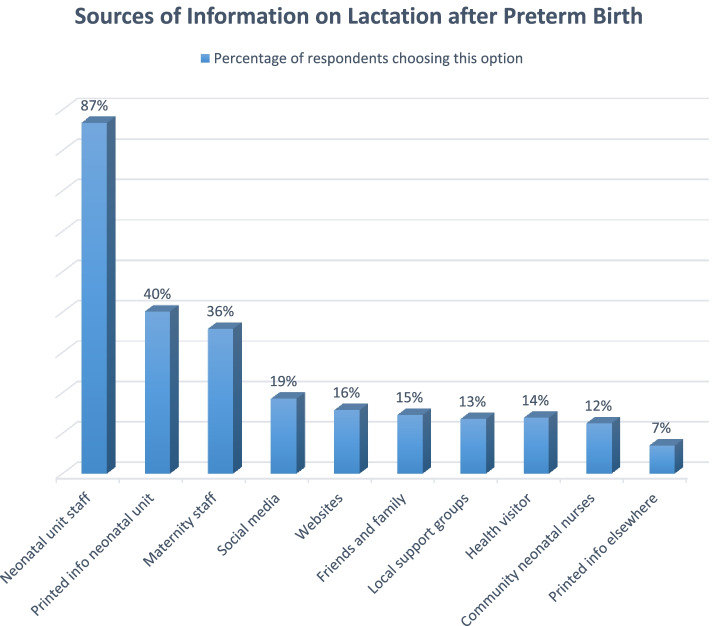
Respondents’ sources of information on lactation for preterm babies. Multiple options could be chosen from a pre-specified list

The questionnaire asked for details about where parents found information about expressing for and breastfeeding their preterm baby. Multiple sources of information were reported, both formal and informal (Fig. 2). In response to predefined options, the majority (87%) of respondents said that they received information from neonatal unit staff and 40% used printed information found in the neonatal unit. 36% received information from maternity staff, such as a midwife, and 19% used social media for this purpose (See Fig. 2).

Free text responses listed a wide variety of social media channels and websites used for information (more than 50). Respondents used Instagram, Twitter, Facebook, Pinterest, YouTube and apps as well as websites as sources of information.

An initial dissemination plan was made using these findings. This focused predominantly on dissemination to clinical staff, particularly neonatal nurses and neonatal infant feeding leads as the key professionals transmitting information to families. A partnership with national charity Best Beginnings is planned to include the trial results, along with wider information about optimal expressing, on printed cards that can be attached to breast pumps in neonatal units. Best Beginnings is a charity providing accessible resources to parents, including parents of sick and preterm infants. A short video is planned to disseminate findings directly to the public, which will utilise the list of social media settings provided by PPI contributors.

#### Influence - future research topics

The distinct area of how to successfully transition from expressing (with gavage feeding, where milk is put directly into the stomach via a tube) to direct breastfeeding was frequently raised. Specific questions for future research are listed in Table 4, in order of frequency raised by respondents. More detail is available in supplementary Table 2 (See Additional file 2 and Table 4).

**Table 4:** Questions for future research on direct breastfeeding in prematurity, from PPI contributors; in order of frequency raised

How to help preterm babies attach to the breast (*n* = 50)	
How to make decisions on the need for gavage supplements when transition to direct breastfeeding has started (*n* = 19)	
When to try direct breastfeeding for the first time (*n* = 19)	
How to transition the breasts from expressing to direct breastfeeding (*n* = 19)	
The effect of “technology” (for example nipple shields, bottles, pacifiers) on the transition to direct breastfeeding (*n* = 17)	
How best to support mothers and partners’ lactation, physical and emotional needs (*n* = 12)	
Personalised likelihood of success at transitioning to any/exclusive direct breastfeeding, and how long the transition is expected to take (*n* = 10)	
What normal behaviour is for a breastfed preterm baby – for example feeding frequency, night waking, crying (*n* = 8)	
Impact of tongue-tie in prematurity (*n* = 1)	

### Stage 3 – charity collaborator

#### Structure

UK national charity Bliss has supported the research project from an early stage. One staff member collaborated on the creation of the funding application, trial protocol and trial documents and supported dissemination of the online questionnaire to parents. A second staff member sits on the Trial Steering Committee. Bliss receives funding for their time, as recommended by an increasing number of research funders.

#### Influence

Input centred on prioritising the mental health and physical needs of parents throughout recruitment and participation in the study, for example, defining an appropriate response to high scores on mental health questionnaires. Bliss also facilitated the very high response rate to the online questionnaire described in Stage 2, through social media dissemination.

### Stage 4 – online panels

#### Structure

A subset of 49 questionnaire respondents who had given consent for further contact and whose infants were under 32 weeks’ gestation at birth were invited in November 2020 to help review trial documents. This included all respondents who were under 25 years old at the birth of their baby or from ethnic minority groups. A formal role description was provided with the offer of associated payment in line with INVOLVE guidance [40].

Two panels were set up using videoconferencing software. Both were scheduled in an evening slot timed with the hope that contributors’ children might be asleep. Panellists were reassured that they could come and go according to their children’s needs. They were asked in advance whether they had any special requirements to maximise participation and sent documents to read in preparation.

Eleven mothers wanted to be involved in the online panels (22% of those invited) and were sent trial documents. Four contributors attended one of two online panels and two further mothers sent written feedback instead (12% of those invited). Two were from Black and other minority ethnic groups and one was under 25 years old. There was a range of gestational age experiences and one mother had experienced neonatal bereavement.

Initial activities were targeted at putting participants at ease, sharing their background and setting ground rules. Each document was discussed in turn and then pre-specified targeted questions were asked on particular terminology and issues that the researcher had experienced while writing the trial protocol. Finally, there was an opportunity for contributors to comment on the trial design and raise other points. Panellists were offered the opportunity to see final trial documents integrating their suggestions after the process was complete, as feedback.

It was also planned to discuss the documents in person one to one with parents currently in a neonatal unit to focus on demographics that were under-represented in the online questionnaire - for example mothers from non-European countries, teenage mothers and mothers from lower socioeconomic backgrounds. Unfortunately, this was not possible because of the SARS-CoV-2 pandemic restricting non-clinical presence in the hospital.

#### Influence – language and focus

Panellists’ feedback led to changes in language used and new documents being created, for example, a short introductory leaflet that would be less overwhelming than the full Participant Information Sheet. Free-text questions were added to the participant entered Case Report Forms to make them feel more accessible and encourage a relationship where the trial participants would feel seen as individuals. Resources were added to the end of each Case Report Form directing people to sources of support related to lactation, mental health and having a preterm baby.

#### Influence – choice of psychometric instrument

Panelists were shown a range of psychometric instruments covering both depression and post-traumatic stress (PTS) reactions. The anxiety instrument had already been chosen (the Spielberger State Trait Anxiety Index) and because a limited number of secondary outcomes could be included within the constraints of the sample size, one further instrument needed to be chosen. There was a clear preference for assessing PTS reactions and for the Post-Traumatic Check List for DSM-5 due to the tone of the language used in the questions. This preference for PTS assessment over depression was because contributors felt that PTS reactions are common after very preterm birth, poorly identified and are a priority for research.

#### Influence – additional training needs

One panellist focused on the need for specific bereavement training for research staff because of the risk of participants experiencing the loss of their baby, or one of their babies for mothers of multiples. All research staff were recommended to undertake bereavement training and some reported the ways in which they used this in talking to bereaved parents within the trial.

### Stage 5 – RCT intervention modification exercise

#### Structure

All respondents who had given consent for further contact were invited in January 2021 to help choose and modify the relaxation recording to be used as the RCT intervention (approximately 250 invitations). The invitation emphasised that people from minority groups were particularly welcomed. A formal role description was provided with an offer of associated payment in line with INVOLVE guidance [40].

Sixty-two people responded to an invitation to be involved in the intervention modification and choice exercise (25% of those invited). Twelve were selected to include a range of gestational ages at birth and to include people from diverse backgrounds and numbers were limited due to PPI payment costs budgeted. Maternal age ranged from 25 to 48 years. One third of the group came from a minority ethnic background and two spoke English as a second language.

Two draft audio files were sent to contributors, both had been used in previous RCTs [41–43]. Contributors were asked for detailed feedback, both open ended and specific and to choose their preferred recording. Once modifications were complete, all contributors were sent feedback on the impact of their involvement.

#### Influence – intervention choice and modification

One recording was clearly preferred by the majority of contributors. The recording script was modified based on three key areas identified by PPI contributors, reducing a feeling of pressure and judgement on having a low milk supply; language and imagery appropriate for sick, immature babies; and language and imagery appropriate for women with traumatic pregnancy and birth experiences.

An example of contributor feedback (emphasis added):

***“The mention of uterus stood out as a trigger word, even now so many years after I had my daughter. I don’t think I would want to be thinking specifically about my uterus as that was linked to trauma. .. There was also a mention of expressing being the number 1 priority, this felt a bit pressured in that moment.”***

### Summary of influence and impact

Key impacts of PPI for this trial were in defining the study outcomes, minimising participant burden and anxiety through trial design, modifying the intervention and informing a dissemination plan. The key impact for future work is providing research questions identified by parents.

Table 5 describes the nature of the influence of PPI on the research project using the Public and Patient Engagement Evaluation Tool (PPEET [44]) for inspiration. The GRIPP2 reporting checklist for PPI reports [45] is also provided as supplementary material (see Additional file 3 and Table 5).

**Table 5:** Areas where PPI influenced the research. Inspired by the Public and Patient Engagement Evaluation Tool (PPEET)

Type of influence	Amount of influence	PPI stage involved	Comment
Choice of population and intervention category	Moderate	Informal PPI (stage 1)	Set before formal PPI started
Choice of trial outcome measures	Large	Informal PPI (stage 1) Online Questionnaire (stage 2) Charity Collaborator (stage 3)	Formal PPI informed the choice of specific outcomes from a range of possibilities and their definitions (for example timepoint assessed)
Areas for exploratory analysis	Moderate	Informal PPI (stage 1) Online Questionnaire (stage 2)	Formal PPI confirmed areas of focus and provided more detail on questions of interest
Trial design – minimising adding to anxiety over milk yield	Large	Online Questionnaire (stage 2) Charity Collaborator (stage 3) Online Panels (stage 4)	Amendments to processes and documents
Trial design – minimising burden for participants	Large	Online Questionnaire (stage 2) Charity Collaborator (stage 3) Online Panels (stage 4)	Amendments to processes and documents. Some areas of tension decided by researchers
Trial modifications in response to challenges	Small	Trial Steering Committee (stage 6)	Consultation. Areas of disagreement decided by researchers
Intervention content	Very Large	Informal PPI (stage 1) Online Questionnaire (stage 2) RCT Intervention Modification Exercise (stage 5)	Iterative changes with detailed PPI input. Some disagreement among participants – final decision by researchers
Dissemination plan	Very Large	Online Questionnaire (stage 2)	Detailed list of vehicles of communication for dissemination provided by PPI, along with assessment of most significant channels
Future research topics	Large	Informal PPI (stage 1) Online Questionnaire (stage 2)	Broad range of questions posed

The Public and Patient Engagement Evaluation Tool has been licensed under a Creative Commons Attribution-NonCommercial-Share Alike 4.0 International License.©2018, Julia Abelson and the PPEET Research-Practice Collaborative. McMaster University. All rights reserved

## Discussion

### Strengths and weaknesses

Patient and Public Involvement had a far-reaching influence on the study during the planning and design phase, which reinforces the importance of PPI at the earliest stages of the research cycle [4]. The online questionnaire format elicited an unexpectedly deep and broad pool of insights to influence trial design and the number of people involved was much larger than other published PPI reports [5, 26, 27]. However a known weakness of using social media to recruit participants [46] is that it is less effective at including marginalised voices, for example, families from ethnic minorities and communities with lower socioeconomic status. This is a frequent theme in PPI, where the people who are easiest to engage are often the most privileged [10, 11]. Thus 95% of online questionnaire respondents were white, whereas only 71% of people giving birth in the UK are white [47].

However, the very large response to the online questionnaire meant that even though harder to reach groups were under-represented proportionally, their voices are still present, the involvement of more than 50 young mothers and 30 mothers from ethnic minorities is a very positive achievement within a PPI setting. Asking about contributor demographics in the online questionnaire meant that minority voices could be preferentially studied and invited for further steps of PPI work. This meant that a third of the panellists and recording exercise contributors were from ethnic minority backgrounds and several were under 25 years old. Despite the strength of this over-sampling approach, diversity of participants was restricted by the data gathered at the questionnaire stage, for example, respondents were not asked about income or education level, or protected characteristics such as disability and sexual orientation, so there are likely areas of under-representation that were not identified or over-sampled. In addition, intersectional communities are less likely to be represented, for example, people from ethnic minority groups who also have low incomes.

There were many aspects of best practice within the PPI process. For example, contributors were adequately compensated (according to INVOLVE guidance [40]) to reduce barriers to involvement and to show the value of their ‘symbolic capital’ [48] as experienced parents with a deep understanding of the trial context. Role descriptions were provided to create a shared understanding of expectations and live interaction was planned to maximise inclusion of this particular population with young children. However around two thirds of those who wanted to participate in online panels did not end up attending which shows the difficulty of providing accessible arrangements for parents of young children, and a range of available times or individual consultations may have been preferable.

A limitation of the PPI design for this project was that the scope and areas of influence of the partnership were predominantly directed and designed by the researcher. There was an unequal balance of power with PPI contributors unable to ensure that their concerns were acted upon in any areas of tension or disagreement. Therefore the level of participation was predominantly ‘consultation’ with some elements of ‘partnership’ rather than higher levels of ‘delegated power’ or ‘citizen control’ [49], these models would require a more fundamental investment in PPI integration into clinical trials units and academic departments.

### Putting findings into context

Some actions resulting from Patient and Public Involvement in this trial are similar to the conclusions from other neonatal and paediatric PPI conducted while planning RCTs [26, 27]. For example, providing participant information in short formats to cater for parents with different information needs, a focus on compassionate language when describing low volumes of maternal milk [26] and giving participants the opportunity to describe their experiences more freely, rather than being constrained to validated questionnaires and numerical responses [27]. However the broad, foundational questions asked in the online questionnaire, and the very large number of responses at an early stage, meant that this PPI process has resulted in a deeper and wider set of transferrable insights than most reports [5, 26, 27]. Thus, in addition to practical changes to trial design, a wide range of future research topics that parents want answers to has been presented, which can be a springboard for the research community.

Much further work needs to be done to answer parents’ questions about how to express milk most effectively and efficiently for their very preterm babies while protecting their mental health, and how to best transition to direct breastfeeding, fulfilling their personal priorities and goals. Future trials seeking these answers can also implement the insights described here to choose meaningful outcomes, minimise parental anxiety and burden and design effective dissemination plans.

### What could be done differently

The PPI process relied heavily on digital literacy and access because of the limitations imposed by the SARS-CoV-2 pandemic. Although this allowed a broader reach and in some cases may have increased accessibility for parents of young children, additional strategies are needed to include those in digital poverty and with communication barriers [10].

Future PPI should be structured to increase the power of PPI participants in the research process. Even without structural changes to integrate shared decision making, contributors can be given more opportunities to challenge the power dynamic by providing anonymous feedback on how the PPI was conducted [23], for example using the PPEET questionnaires [44].

Any weaknesses of this process were partially the result of Patient and Public Involvement taking place without initial funded resource, and partly due to the inexperience of the researcher, both are acknowledged barriers to high quality PPI within doctoral research [5], although seen at all levels [4]. University Departmental PPI networks and support systems could help overcome both resource pinch points and provision of expertise.

## Conclusions

Investing time, expertise and financial resource into Patient and Public Involvement at an early stage can have a very significant impact on research, including randomised controlled trials. This may be particularly important in a high stress environment such as after very preterm birth and where the condition under study (lactation) is influenced by complex emotional and physical contributory factors. This report also demonstrates that under-representation of rarely reached groups is not inevitable within Patient and Public Involvement.

Much can be learnt from reporting Patient and Public Involvement processes and findings in full. This report may help other researchers plan effective and iterative PPI for their own research. Lessons learned can be translated to future research in relation to the questions and outcomes that are important to parents in this field, as well as effective dissemination pathways. In addition, this detailed report emphasises to the academic community as well as to PPI contributors themselves, the high value and importance of their involvement.

## Supplementary Information

**Additional file 1: Supplementary Table 1.** Detailed list of questions for future research on breastmilk expression. A table listing detailed questions for future research on breastmilk expression, submitted by 675 respondents to an online questionnaire for parents of premature babies [50].

**Additional file 2: Supplementary Table 2.** Detailed list of questions for future research on direct breastfeeding for preterm infants. A table listing detailed questions for future research on direct breastfeeding, submitted by 675 respondents to an online questionnaire for parents of premature babies.

**Additional file 3.** GRIPP2 short form reporting checklist. A completed GRIPP2 checklist (Guidance for Reporting Involvement of Patients and the Public).

## Data Availability

Not applicable.

## Abbreviations

DSM: Diagnostic and Statistical Manual of Mental Disorders
GRIPP2: Guidance for Reporting Involvement of Patients and the Public (second revision)
MOM: mothers’ own milk
PPEET: Public and Patient Engagement Evaluation Tool
PPI: Public and Patient Involvement
PTS: Post traumatic stress
RCT: Randomised controlled trial
SARS-CoV-2: Severe Acute Respiratory Syndrome Coronavirus 2
