# Supplementary material for: A report on parent involvement in planning a randomised controlled trial in neonatology and lactation – insights for current and future research
Source: Int Breastfeed J. 2022 Sep 14;17:69. doi: 10.1186/s13006-022-00509-1 (PMC9472727; doi:10.1186/s13006-022-00509-1)
Supplement: Supplementary file 1 — Additional file 1: Supplementary Table 1. Detailed list of questions for future research on breastmilk expression. A table listing detailed questions for future research on breastmilk expression, submitted by 675 respondents to an online questionnaire for parents of premature babies [50]. [file 13006_2022_509_MOESM1_ESM.docx]

**Supplementary Table 1: Detailed list of questions for future research on breastmilk expression, from PPI contributors, in order of frequency mentioned**

| How frequently to express, including whether night expressions are important for milk supply and whether frequency can be reduced after milk supply is established. Some respondents specifically wanted to know a ‘realistic’ frequency of expressing to target, stating that the advised 8-10 times a day was impossible or unrealistic |
| --- |
| How much milk should be targeted/expected, how this changes over time and whether there are particular time windows that are most important to establish a good milk supply |
| How best to relax/feel comfortable or happy while expressing milk |
| How long to express for at each session – whether this should be targeted by the volume expressed or sticking to a particular length of time or another measure; how long to express for if there is no resulting milk |
| Whether there are particular foods that increase or decrease yield |
| The impact of milk storage on quality and composition (for example whether it’s better for babies to be fed fresh or refrigerated milk than frozen, how length of freezing affects the milk, in what way to store milk for optimal use) |
| What is the optimal time interval between expressing sessions or pattern of expressing over 24 hours |
| How to minimise and manage over-supply |
| What their personalised likelihood is of being able to express good volumes of milk, for example considering the birth gestation of their baby, medical problems experienced by the mother around the birth, mode of birth, delays in starting expression and early volumes that can be expressed |
| Whether hand expressing is better than an electric pump and the optimal time of switching between the two |
| Whether any particular type of pump is better than others |
| How to most effectively induce ‘let down’ (the milk ejection reflex) |
| Whether medications and herbal medicines increase yield |
| The impact of delayed first expression |
| Whether massaging the breast while expressing increases yield and/or reduces mastitis |
| How to reduce the occurrence of blocked milk ducts and mastitis |
| Whether different forms of sterilisation have an effect on milk quality and composition (for example chemical contamination of milk after use of chemical sterilisation) and whether sterilisation of equipment is needed |
| Whether expressing at the cot side increases yield compared to an expressing room |
| Whether seeing or smelling things that reminded them of their baby increases yield |
| Whether antenatal expressing increases yield |
| The personalised normal time period for milk to ‘come in’ (lactogenesis II; for example considering the birth gestation of their baby and birth method) |
| Whether drinking more fluid increases milk supply and if so, what to target |
| Whether warming the breast increases yield |
