# Supplementary material for: A report on parent involvement in planning a randomised controlled trial in neonatology and lactation – insights for current and future research
Source: Int Breastfeed J. 2022 Sep 14;17:69. doi: 10.1186/s13006-022-00509-1 (PMC9472727; doi:10.1186/s13006-022-00509-1)
Supplement: Supplementary file 2 — Additional file 2: Supplementary Table 2. Detailed list of questions for future research on direct breastfeeding for preterm infants. A table listing detailed questions for future research on direct breastfeeding, submitted by 675 respondents to an online questionnaire for parents of premature babies. [file 13006_2022_509_MOESM2_ESM.docx]

**Supplementary Table 2: Detailed list of questions for future research on direct breastfeeding for preterm infants, from PPI contributors, in order of frequency mentioned**

| How to help preterm babies latch to the breast, including best positions to use, how to encourage a sleepy baby to suck and modifications for babies with medical needs such as oxygen |
| --- |
| During the transition from gavage feeding to oral feeding, how to decide when and how long to directly breastfeed for at each feed, and then how to decide whether and how much to give as a supplement |
| The effect of nipple shields, bottles, dummies and other assistive strategies (including supplemental nursing systems and cups) on the successful transition to direct breastfeeding |
| When to start the process of trying to directly breastfeed for the first time (for example what gestation/age, weight or looking out for what cues from the baby) |
| How to transition the breasts from expressing to breastfeeding, including dealing with engorgement [milk building up in the breast tissue, causing pain and increasing the risk of breast inflammation and mastitis] when the baby is breastfeeding less than the previous pumped milk volumes and deciding how much to express in addition to direct breastfeeding |
| What their personalised likelihood of success is for transitioning to any/exclusive direct breastfeeding, for example considering the birth gestation of their baby, the baby’s medical problems, twins versus a single baby and how much milk they can express |
| When responsive feeding can be used (for example what gestation/age, weight or what evidence is required from behaviour/weight pattern) |
| What normal behaviour is for a breastfed preterm baby – for example feeding frequency, night waking, crying |
| Understanding how long the transition from gavage to oral feeding takes and what might happen to the baby’s weight during the transition |
| How best to support mothers and partners, in terms of physical and emotional needs, in establishing breastfeeding in hospital and after discharge home |
| What is the best strategy for getting extra calories into a breastfeeding infant where required (for example breastmilk fortifier, high energy formula, with what assistive technology) |
| How best to relax during the process of transitioning to direct breastfeeding |
| Whether to express in addition to breastfeeding even if the baby is solely breastfeeding (for example with a late preterm baby – whether additional expressing is helpful for the long term milk supply) |
| Where the family want to mixed feed (use infant formula and breastmilk), what factors will maximise the likelihood of being able to continue doing so long-term (for example, should formula and breastmilk feeds be kept separate, the impact of bottle use on direct breastfeeding, whether there is a minimum amount of breastfeeding or expressing required) |
| Whether specialist infant feeding support is better than routine infant feeding support |
| When to treat for tongue tie |
