# Supplementary material for: A report on parent involvement in planning a randomised controlled trial in neonatology and lactation – insights for current and future research
Source: Int Breastfeed J. 2022 Sep 14;17:69. doi: 10.1186/s13006-022-00509-1 (PMC9472727; doi:10.1186/s13006-022-00509-1)
Supplement: Supplementary file 3 — Additional file 3. GRIPP2 short form reporting checklist. A completed GRIPP2 checklist (Guidance for Reporting Involvement of Patients and the Public). [file 13006_2022_509_MOESM3_ESM.docx]

**GRIPP2 short form reporting checklist:**

| Section and topic | Item |
| --- | --- |
| 1: Aim | For parents of preterm babies to guide the choice of research question, study design, content of participant-facing elements, to provide oversight of the trial and collaborate in dissemination of findings. |
| 2: Methods | Multi-stage methods: Online questionnaire open to all and advertised via a PPI charity collaborator. Responses from 675 parents. Online PPI panels – invitations sent to questionnaire respondents with over sampling of under-reached populations. Six parents took part. Interactive PPI exercise informing study intervention design – invitation sent out to all questionnaire respondents with choice of contributors made with reference to the diversity of their background and experience. 12 parents took part. One contributor invited to join trial steering group, along with PPI charity collaborator, who was also consulted at all stages of trial design. |
| 3: Study results | PPI:   - contributed to choice of study question/primary and secondary outcomes, and provided a range of further research priorities for the future - resulted in changes to study design and participant facing documents, particularly to the modification of the study intervention itself - provides oversight of the study |
| 4: Discussion and conclusions | PPI increased the relevance of the study question and the likelihood of good recruitment and retention levels. PPI was important in maximising the utility of the intervention itself and minimising as far as was possible the potential for the intervention to make participants anxious or over-burdened.  PPI was fairly one directional, with limited possibilities for PPI contributors to ensure that their views had been heard and acted upon or to become involved in the day to day work of the research. |
| 5: Reflections/critical perspective | Some efforts were made to ensure that a diverse range of parents were involved, by over-sampling under-reached groups and selecting contributors with conscious reference to the diversity of their experiences. However reliance on digital methods due to the SARS-CoV2 pandemic limited the extent to which the some under-reached groups were involved. For example, there were few PPI contributors from socioeconomically deprived backgrounds and with non-European first languages. Future projects could also seek to embed the PPI collaboration at an earlier stage and with meaningful bidirectional communication and control, providing training for contributors and researchers if needed. |
